# 9‐Cyano‐10‐telluriumpyronin Derivatives as Red‐light‐activatable Raman Probes

**DOI:** 10.1002/asia.202201086

**Published:** 2022-12-13

**Authors:** Minoru Kawatani, Spencer J. Spratt, Hiroyoshi Fujioka, Jingwen Shou, Yoshihiro Misawa, Ryosuke Kojima, Yasuteru Urano, Yasuyuki Ozeki, Mako Kamiya

**Affiliations:** ^1^ Department of Life Science and Technology Tokyo Institute of Technology Kanagawa 226-8501 Japan; ^2^ Graduate School of Medicine The University of Tokyo Tokyo 113-0033 Japan; ^3^ Department of Electrical Engineering and Information Systems The University of Tokyo Tokyo 113-8656 Japan; ^4^ Graduate School of Pharmaceutical Sciences The University of Tokyo Tokyo 113-0033 Japan; ^5^ Living Systems Materialogy (LiSM) Research Group International Research Frontiers Initiative (IRFI) Tokyo Institute of Technology Kanagawa 226-8501 Japan

**Keywords:** Raman spectroscopy, Stimulated Raman scattering, Tellurium, Dyes/Pigments, Photoactivation

## Abstract

Photoactivatable fluorescence probes can track the dynamics of specific cells or biomolecules with high spatiotemporal resolution, but their broad absorption and emission peaks limit the number of wavelength windows that can be employed simultaneously. In contrast, the narrower peak width of Raman signals offers more scope for simultaneous discrimination of multiple targets, and therefore a palette of photoactivatable Raman probes would enable more comprehensive investigation of biological phenomena. Herein we report 9‐cyano‐10‐telluriumpyronin (9CN‐TeP) derivatives as photoactivatable Raman probes whose stimulated Raman scattering (SRS) intensity is enhanced by photooxidation of the tellurium atom. Modification to increase the stability of the oxidation product led to a julolidine‐like derivative, 9CN‐diMeJTeP, which is photo‐oxidized at the tellurium atom by red light irradiation to afford a sufficiently stable oxidation product with strong electronic pre‐resonance, resulting in a bathochromic shift of the absorption spectrum and increased SRS intensity.

## Graphical abstract

We developed 9CN‐TeP derivatives having a tellurium atom at the 10th position of xanthene as a reaction point of photooxidation. These red‐light‐activatable Raman probes show a bathochromic shift in the absorption spectrum and enhanced stimulated Raman scattering (SRS) intensity after red light irradiation.

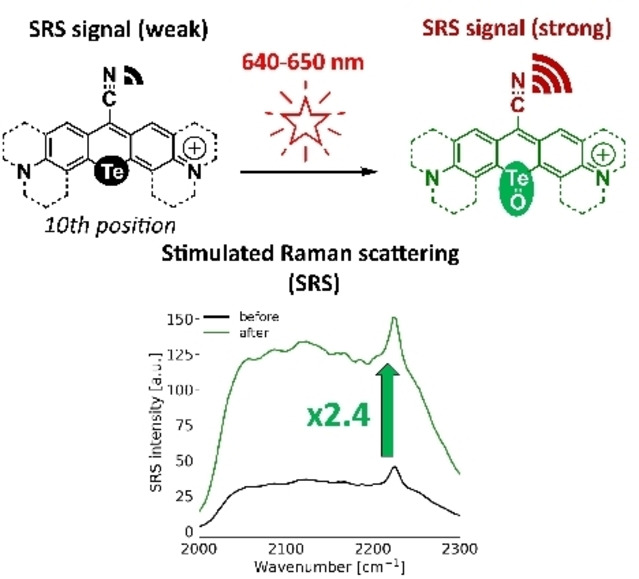

## Introduction

Optical imaging of biological samples, especially fluorescence imaging, has been widely used in the fields of biology and medicine to track the dynamics of cells or biomolecules with high spatiotemporal resolution, and many photoactivatable fluorescence probes whose fluorescence can be turned ON upon light irradiation under microscopy have been synthesized.[^1^, ^2^, ^3^, ^4^, ^5^] Indeed, a palette of photoactivatable dyes that can be activated at various wavelengths from ultraviolet (UV) to the visible light range[^6^, ^7^] has been developed, enabling multiple targets to be tracked simultaneously. However, the absorption and emission spectra of fluorescent molecules are rather broad, and thus the number of biological targets that can be detected simultaneously using different optical windows is limited.

Raman imaging is an attractive alternative approach to visualize biomolecules or biological responses by detecting molecular vibrations. Since the Raman spectral width at half maximum is ∼50 times narrower than that of fluorescence, Raman signals offer much greater scope for simultaneous discrimination of multiple targets.[^8^, ^9^] On the other hand, spontaneous Raman signals are very weak, which limits many bio‐imaging applications. However, recent progress in non‐linear optical imaging techniques including stimulated Raman scattering (SRS) is overcoming this limitation. Since SRS signals can be observed orthogonally to fluorescence, and we can significantly enhance the Raman signal, this approach is very promising for multiplex biological imaging.[^8^, ^9^] In addition, the Raman signal can be further enhanced by a factor of >10^4^ under an electronic pre‐resonance (EPR) condition, where the molecular absorption wavelength of the fluorophore approaches the pump beam wavelength.^[10]^ Recently, 9‐cyanopyronin derivatives have been reported as SRS‐tailored dyes for Raman tagging.[^11^, ^12^] The red to near‐infrared absorption of 9‐cyanopyronins amplifies their SRS signals through EPR upon exposure to a typical SRS pump beam (800–900 nm). The strong SRS signal in the cell‐silent region (1800–2800 cm^−1^)^[13]^ derived from the nitrile group at the 9th position further increases the signal‐to‐background ratio. Moreover, expansion of the color palette can be rationally achieved by isotopic substitution of nitrile groups and structural expansion.^[12]^

**Figure 1:**
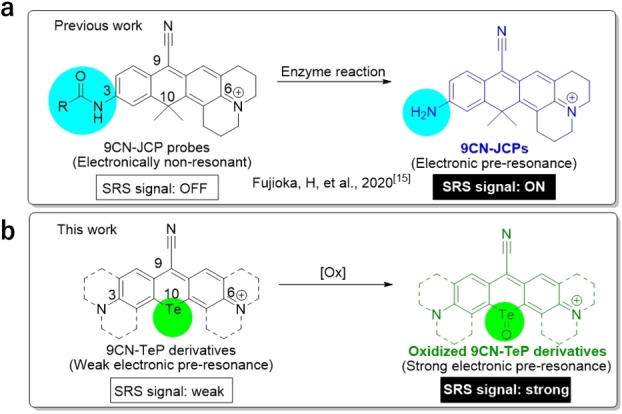
9‐Cyanopyronin probes reported by Fujioka et al. (a), and developed in the present work (b).

We have recently established a molecular design strategy for activatable Raman probes for detecting enzyme activities by means of SRS imaging.^[14]^ SRS signal activation in response to an enzyme activity is achieved by controlling the resonance Raman effect through changes in the absorption spectra. When an enzyme substrate moiety at the 3rd position of 9‐cyanopyronin is cleaved by the target enzyme, a large bathochromic shift is induced, resulting in SRS signal activation via enhancement of the EPR (Figure 1a). Building on these results, we considered that we could develop a range of activatable Raman probes by designing molecules whose absorption wavelengths become longer and closer to the wavelength of the pump laser in response to specific reactions. In the present work, we set out to test this idea by designing photo‐activatable Raman probes that would show a bathochromic shift in the absorption spectrum upon photo‐irradiation. For this purpose, we focused on the fact that rhodamine derivatives with a tellurium atom at the 10th position (TeRhodamines) show a bathochromic shift of more than 60 nm upon oxidation of tellurium with an oxidant or by photo‐oxidation.[^15^, ^16^, ^17^, ^18^] Specifically, we designed and synthesized a new class of 9‐cyanopyronin derivatives with tellurium at the 10th position, i. e., 9‐cyano‐10‐telluriumpyronin (9CN‐TeP) derivatives. These 9CN‐TePs exhibit a sufficient SRS signal in the cell‐silent region (Figure 1b). Furthermore, 9CN‐TePs underwent a large bathochromic absorption shift upon the oxidation of tellurium. Among the synthesized derivatives, 9CN‐diMeJTeP exhibited sufficiently high post‐oxidation stability, and the SRS signal intensity could be photoactivated (2.4‐fold) by red‐light irradiation as a result of the 67 nm bathochromic shift. These results indicated that the 10th position can be used as a key reaction point for designing activatable Raman probes, and that 9CN‐diMeJTeP can work as a red‐light‐activatable Raman dye for SRS imaging.

## Results and Discussion

Inspired by TeRhodamines, whose tellurium atom has been reported to be oxidized by photoirradiation with an accompanying bathochromic shift,[^17^, ^18^] we initially designed and synthesized 9CN‐TePY, a pyronin Y derivative with tellurium at the 10th position. Te‐xanthone was synthesized by the reported method,^[17]^ followed by reduction with lithium aluminum hydride and oxidation with *p*‐chloranil to afford TePY, then the nitrile group at the 9th position was introduced to obtain 9CN‐TePY (Scheme S1).

**Figure 2:**
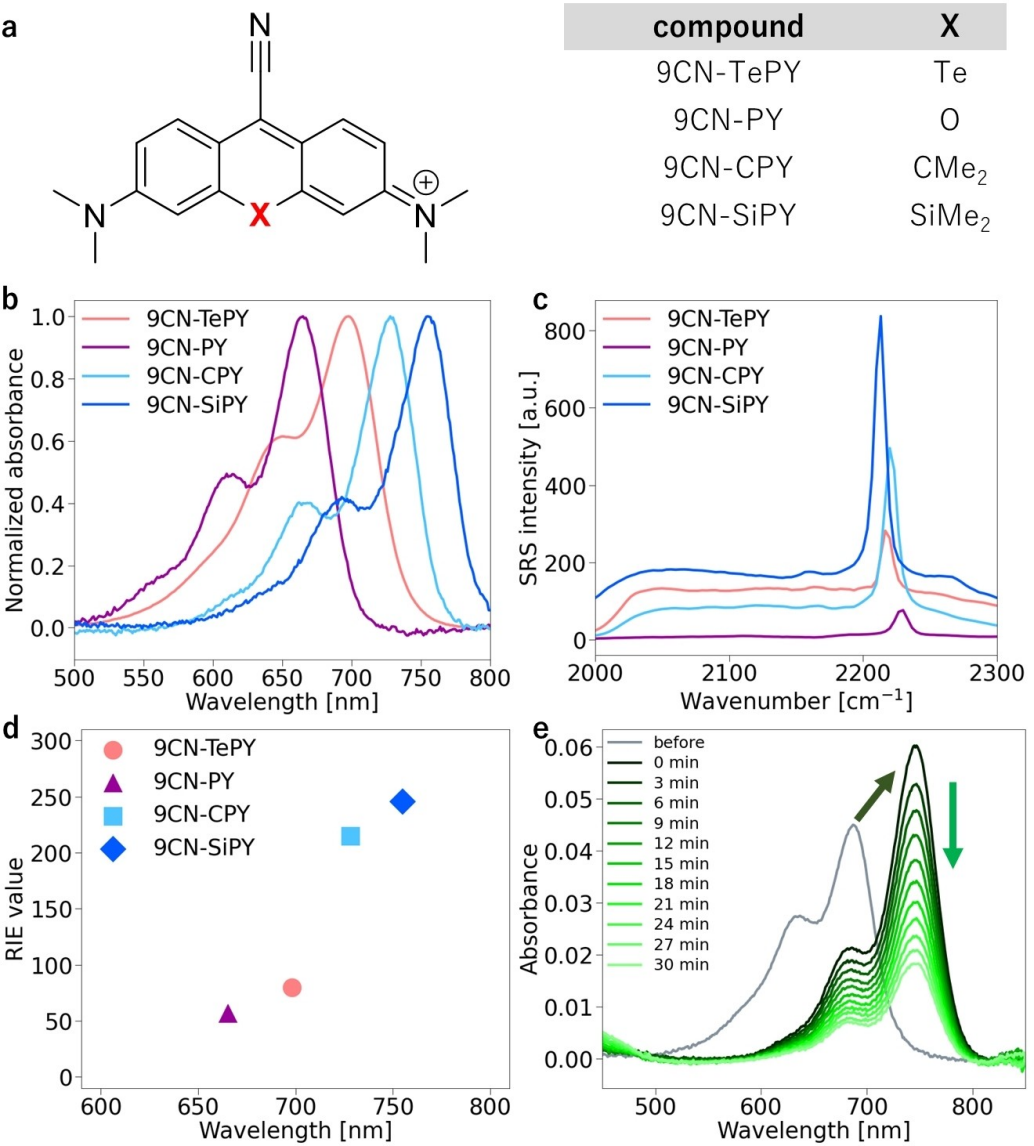
Characterization of 9CN‐TePY versus other 9‐cyano pyronin Y (9CN‐PY) derivatives. (a)–(d) Photophysical properties. (a) Chemical structure of 9CN‐PY derivatives. (b) Normalized absorption spectra in DMSO. (c) SRS intensity of 1 mM 9CN‐PY derivatives in DMSO. (d) RIE of 9CN‐PY derivatives in DMSO versus their maximum absorption wavelength. (e) Change in absorption spectra of 9CN‐TePY after oxidation by NaOCl. Solvent: PBS, concentration: 0.5 μM (9CN‐TePY), 2 μM (NaOCl). Absorption spectra were measured before oxidation (before) and at 3 min intervals after addition (0–30 min).

9CN‐TePY showed a maximum absorption wavelength of 698 nm, which is 28 nm longer than that of 9‐cyano pyronin Y (9CN‐PY), an O‐core derivative (Table 1, Figure 2a, 2b). The SRS spectrum showed a strong single peak at 2217 cm^−1^ in the cell‐silent region, as observed with other 9‐cyano‐10‐chalcogen‐pyronin derivatives^[19]^ (Table 1, Figure 2c). The relative Raman intensity versus EdU (RIE),^[20]^ which is commonly used as an index of the brightness of Raman probes, was 80, approaching that of other EPR‐SRS dyes available for bioimaging, such as MARS 2238 (RIE=108).[^9^, ^12^] We found that the RIE values of 9CN‐TePY and other 9CN‐PY derivatives increase as the absorption wavelength gets longer. This is consistent with an EPR effect, as previously shown with other dyes similar to 9‐cyanopyronin derivatives (Figure 2d).^[11]^ Next, we examined the stability of 9CN‐TePY. During incubation over a week, 9CN‐TePY underwent degradation at a similar rate to other 9CN‐pyronin Y derivatives (Figure S1a).

**Table 1:**
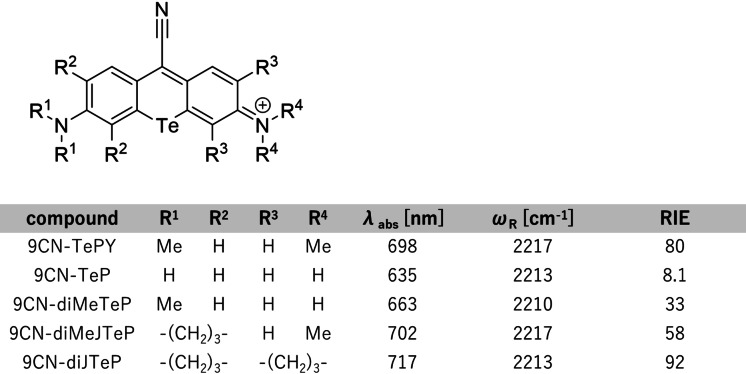
Chemical structures and photophysical properties of 9CN‐TeP derivatives. Photophysical properties were measured in DMSO containing 10% DMF as a cosolvent. λ_abs_: Absorption maximum. *ω*_R_: Raman shift of the C≡N vibrational mode.

To examine the photophysical properties of the oxidation product of 9CN‐TePY, hypochlorite was added to 9CN‐TePY solution in PBS. The absorption spectrum quickly shifted to longer wavelength, followed by a steady decrease in the absorbance (Figure 2e). LC–MS analysis of the reaction solution showed that 9CN‐TePY was converted to a xanthone derivative (Figure S2).

**Figure 3:**
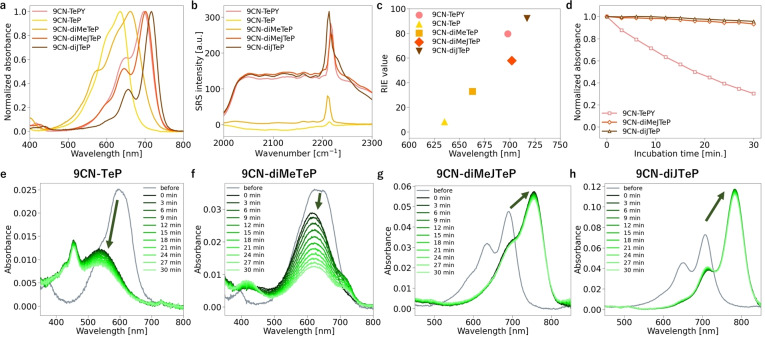
Characterization of 9CN‐TeP derivatives. (a)–(c) Photophysical properties of 9CN‐TePY compared with other 9CN‐TeP derivatives. (a) Normalized absorption spectra in DMSO. (b) SRS intensity of 1 mM 9CN‐TeP derivatives in DMSO. (c) RIE of 9CN‐TeP derivatives in DMSO versus maximum absorption wavelength. (d)–(h) Change in absorption spectra of 9CN‐TeP derivatives after oxidation by NaOCl. (d) Time course of the bathochromic shift of peak absorbance after oxidation, extracted from (f)–(h). No bathochromic shift was observed for 9CN‐TeP and 9CN‐diMeTeP. (e) 9CN‐TeP, (f) 9CN‐diMeTeP, (g) 9CN‐diMeJTeP, (h) 9CN‐diJTeP. Solvent: PBS, concentration: 0.5 μM (9CN‐TeP derivatives), 2 μM (NaOCl). Absorption spectra were measured before oxidation (before) and at 3 min intervals after addition (0–30 min).

While 9CN‐TePY shows promising properties in terms of the relatively strong SRS signal and the drastic change in the absorption spectrum upon oxidation, we could not observe the SRS spectrum of the oxidation product due to its instability. Thus, we next focused on structural evolution of 9CN‐TePY to find more stable derivatives. For this purpose, we designed and synthesized 9CN‐TeP derivatives with an amino group or julolidine‐like structure at the 3rd or 6th position, because it has been reported that *N*‐substitution of pyronins affects their stability[^21^, ^22^] (Scheme S2–S5). The absorption peak wavelengths of the derivatives with an amino group (9CN‐TeP and 9CN‐diMeTeP) were much shorter than that of 9CN‐TePY, while those of the derivatives with a julolidine‐like structure (9CN‐diMeJTeP and 9CN‐diJTeP) showed longer wavelengths compared to 9CN‐TePY (Table 1, Figure 3a). Strong SRS spectra were also observed, as in the case of 9CN‐TePY (Figure 3b). The relatively high background signals from 9CN‐TePY, 9CN‐diMeJTeP and 9CN‐diJTeP might be due to non‐linear optical processes, such as two‐photon absorption,^[23]^ or possible by‐product formation in the photoreaction. The RIE of each derivative tended to increase as peak wavelength became longer (Figure 3c). Compared to 9CN‐TePY, 9CN‐TeP and 9CN‐diMeTeP were less stable, while 9CN‐diMeJTeP and 9CN‐diJTeP showed greater stability during incubation (Figure S1b). These results are consistent with previous reports indicating that pyronin derivatives with higher electrophilicity have lower stability in aqueous solution.[^12^, ^14^]

To observe the absorption spectra of the oxidized forms, hypochlorite was added to the dye solutions in PBS. 9CN‐diMeTeP, 9CN‐diMeJTeP and 9CN‐diJTeP each showed a bathochromic shift of about 60 nm after oxidation, although 9CN‐diMeTeP decomposed rapidly, like 9CN‐TePY. Fortunately, 9CN‐diMeJTeP and 9CN‐diJTeP remained stable even after oxidation, suggesting that the introduction of the julolidine‐like structure was effective to improve the stability of the oxidation product (Figure 2e, 3d–h). Decomposition was observed only when the concentration of hypochlorite was increased (Figure S3). Based on these results, we selected 9CN‐diMeJTeP and 9CN‐diJTeP for further study.

**Figure 4:**
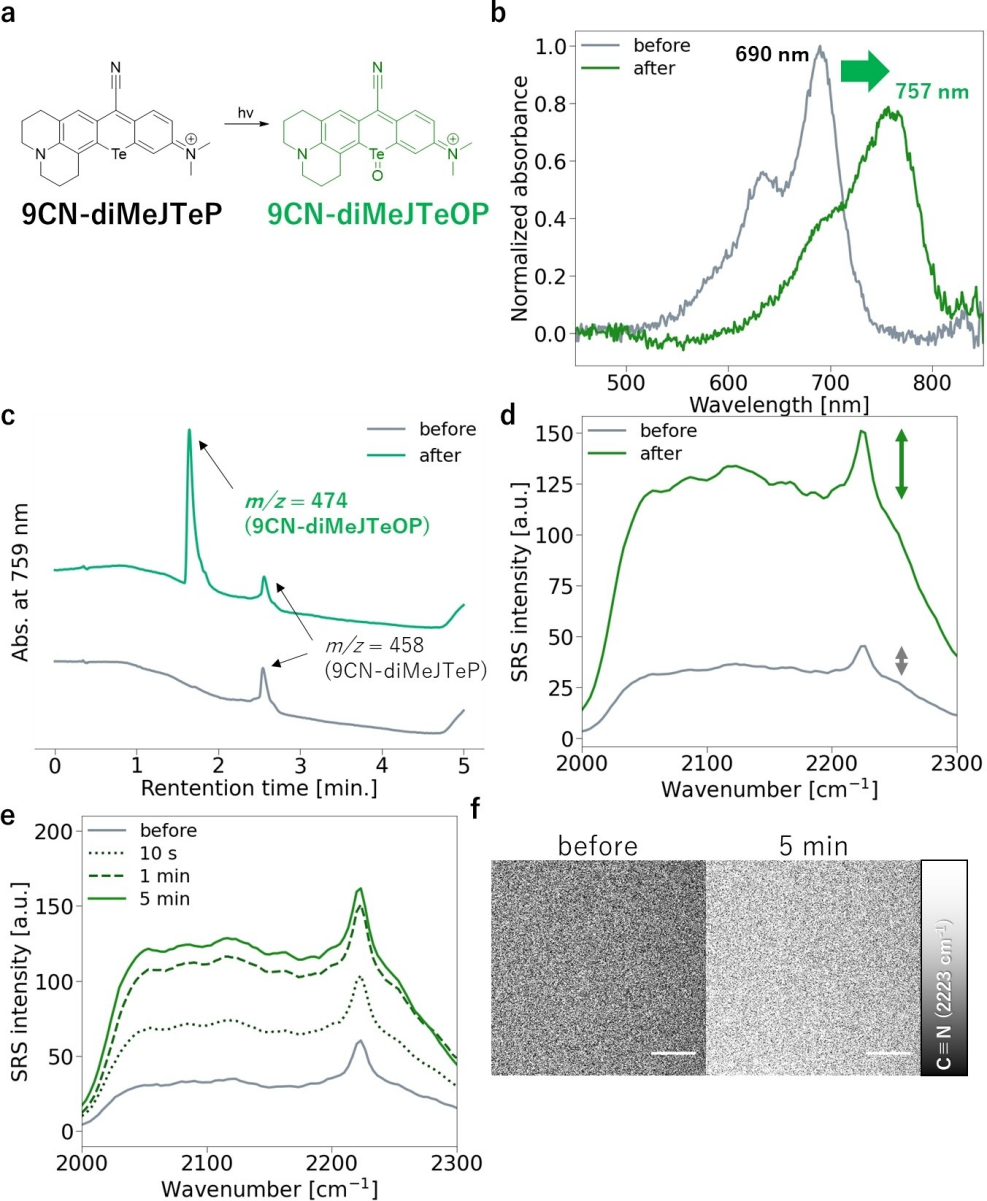
Photoactivation of 9CN‐diMeJTeP. (a) Reaction scheme of photooxidation. (b) Change in absorption spectra upon photooxidation, and (d) change in SRS spectra. (c) LC–MS analysis of the photoproduct. Absorbance at 759 nm was monitored. (e)–(f) *in situ* photoactivation of 9CN‐diMeJTeP on SRS microscopy by 640 nm CW light. Bars indicate the peak intensity. (e) Change in SRS spectra upon photoactivation. (f) SRS image of 9CN‐diMeJTeP solution at 2223 cm^−1^. Scale bars, 10 μm. Solvent: 20 mM sodium phosphate buffer pH 2.0, concentration of dye for photoreaction: 100 μM, DMF: 10%. Absorption spectra were measured by diluting the 10 μL reaction solution to 2.5 mL with 20 mM sodium phosphate buffer pH 2.0 (final concentration: 0.4 μM). Photoreaction for (b)–(d): 45 mW/cm^2^, xenon light through a 650/10 nm bandpass filter. Photoreaction for (e)–(f): 20 mW 640 nm laser light was introduced during observation.

Considering that TeRhodamines are self‐oxidized upon excitation, a similar photooxidation reaction could occur with 9CN‐TeP derivatives. We anticipated that a bathochromic shift of the absorption spectrum would be induced by the excitation light through self‐sensitization of 9CN‐TeP derivatives, resulting in an increase of the SRS signal intensity due to enhancement of EPR (Figure 4a, Figure S4a). We initially examined the photoreaction of 9CN‐TeP derivatives under an acidic condition (20 mM sodium phosphate buffer pH 2.0) in accordance with the previous report.^[17]^ Red‐light (650 nm from a xenon lamp) irradiation of 9CN‐diMeJTeP or 9CN‐diJTeP in acidic buffer caused a bathochromic shift of about 70 nm in the absorption spectrum (Figure 4b, Figure S4b). LC–MS analysis of the reaction solution revealed a photoproduct that was identified as the oxidized compound (9CN‐diMeJTeOP: *m/z*=474 (Figure 4c) or 9CN‐diJTeOP: *m/z*=526 (Figure S4c), respectively). Red‐light irradiation of 9CN‐diMeJTeP induced a 2.4‐fold increase in SRS peak intensity when compared with non‐irradiated solution (Figure 4d). An increase in the background signal was also observed, possibly due to multiple competitive electronically enhanced pathways, as mentioned above. On the other hand, the SRS spectra of 9CN‐diJTeP in aqueous solution showed no obvious peak in the cell‐silent region, but there was a high background signal compared with 9CN‐diMeJTeP. Moreover, the SRS intensity was reduced after red‐light irradiation (Figure S4d). Although the reason for these unexpected results in SRS measurements of 9CN‐diJTeP is still under investigation, we nevertheless selected 9CN‐diMeJTeP for further study as a promising candidate for a red‐light‐activatable Raman probe.

Photoactivation of 9CN‐diMeJTeP *in situ* on an SRS microscope was conducted. Irradiation of 9CN‐diMeJTeP in acidic buffer with 640 nm laser light on an SRS microscope induced a rapid increase in the SRS signal (Figure 4e, 4 f). On the other hand, we did not observe any photoactivation when pump and Stokes lights were introduced onto the sample for SRS observation (Figure S5).

Next, we examined the photoactivation of 9CN‐diMeJTeP in neutral solution to evaluate the reaction under a more physiological condition. We observed a bathochromic shift of the absorption wavelength of 9CN‐diMeJTeP after photooxidation in PBS (pH 7.4), and the photoproduct showed good stability (Figure S6). Encouraged by this result, we measured the SRS spectral change upon red‐light irradiation. A rapid increase of the SRS signal intensity was observed after light irradiation at 640 nm (Figure S7). These data suggest that red‐light activation of 9CN‐diMeJTeP also works under neutral conditions, and therefore the probe should be suitable for biological applications.

## Conclusion

We have developed novel photoactivatable Raman probes, 9CN‐TeP derivatives, which contain a tellurium atom at the 10th position of the xanthene core as a reaction site for oxidation. Although a large bathochromic shift was induced by the oxidation for 9CN‐TeP derivatives, the stability of the oxidation products was relatively low. However, structural evolution afforded a more stable derivative 9CN‐diMeJTeP. The SRS intensity of 9CN‐diMeJTeP was photoactivated by red light, and we confirmed activation *in situ* by 640 nm laser light under an SRS microscope. Since observation light for SRS imaging (pump/Stokes light) did not induce photoactivation, we concluded that 9CN‐diMeJTeP would be suitable as a red‐light‐activatable Raman probe to investigate the spatiotemporal dynamics of target biomolecules, in a similar manner to widely used fluorescence probes.[^24^, ^25^] Unlike fluorescence imaging, SRS imaging has the great advantage that the excitation light required for observation does not occupy the wavelength range required for photoactivation. Therefore, multiplexed detection is possible, as well as orthogonally multiplexed photoactivation in combination with other caged or photoswitchable Raman probes that can be activated by UV to yellow light.[^26^, ^27^, ^28^] Furthermore, since oxidation of the tellurium atom could be induced by hypochlorite, it should be possible to develop an activatable Raman probe based on 9CN‐TeP structure to target hypochlorite, an important reactive oxygen species.

In addition to the previously reported reaction point at the 3rd position,^[14]^ our present strategy, based on a reaction point at the 10th position of the xanthene structure, is expected to greatly expand the scope of molecular design of Raman probes. One possible concern is the relatively low stability of the oxidation products of 9CN‐TePs, and thus further improvement of the stability would be beneficial for biological applications. It has been shown that the stability of TeRhodamines can be improved by introducing Me groups into the benzene moiety, thereby suppressing inter‐ or intramolecular nucleophilic attack of oxygen from 10th position of the oxidized form.^[17]^ A similar reaction could also occur in the case of 9CN‐TeP derivatives, so it should be feasible to further increase the stability of the 9CN‐TeP oxidation products by introducing substituents at 1st or 8th position of xanthene that would block attack on the 9th position. Such Raman probes are expected to enable SRS observation to track multiple targets in live cells and tissues.

## Experimental Section

***SRS microscopy***. For SRS imaging, a Ti: sapphire laser (Coherent, Mira900D) provides a pump pulse with 843.26 nm central wavelength and 76 MHz repetition rate. A custom ytterbium‐doped fiber laser (YDFL) system provides Stokes pulses with a tunable central wavelength from 1014 nm to 1046 nm (corresponding to the wavenumber region of 2000–2300 cm^−1^) and 38 MHz repetition rate. The Ti: sapphire laser and the YDFL are synchronized by means of a feedback circuit. For imaging, the pump light and Stokes light are spatially combined by a dichroic mirror and temporally aligned by adjusting the time delay line, then led to an inverted video‐rate point‐scanning microscope equipped with a 640 nm continuous‐wave laser for *in situ* photoactivation of 9CN‐diMeJTeP. A resonant galvanometric scanner operating at 8 kHz and an ordinary galvanometric scanner are used to acquire video at 30 frame/s, and water‐immersion objective lenses (Olympus, 60x, N.A.=1.2) are used for light focusing and collection. The transmitted pump light is filtered out and detected by a Si photodiode, the output of which is further demodulated by a lock‐in amplifier to generate the SRS signal.

***SRS spectral measurements and SRS imaging***. SRS spectra were obtained with the SRS microscope as described above. All samples were held in imaging chambers consisting of two glass coverslips (Matsunami, C218181, C024361) by using an imaging spacer (Merck, Grace Bio‐Labs SecureSeal imaging spacer, GBL654002‐100EA, 0.12 mm thickness). The chamber was filled with dye solution containing PMMA beads (Sekisui, Thechpolymer SSX‐110, average particle size: 10 μm) to facilitate finding the position of the solution and sealed with nail polish. Each point of the SRS spectrum was acquired by averaging the pixel values of 5 frames at a certain wavenumber. Imaging area: 80 μm×80 μm×2 μm, scanning point: 450 nm×450 nm×2 μm, pixel dwell time: 70 ns at the center of the FOV (note that the pixel dwell time depends on the x position because of the sinusoidal motion of the resonant scanner operating at 8 kHz).

***Absorption spectral measurements***. Absorption spectra were obtained with a UV‐2450 UV–Vis spectrometer (Shimadzu). Probes were dissolved in DMF (Super dehydrated, FUJIFILM Wako Pure Chemical) to obtain stock solutions.

***RIE measurements***. 100 mM EdU solution in DMSO was used as an external standard. SRS spectra from 1 mM 9‐cyanopyronin derivatives in DMSO were used to calculate RIE.

***LC–MS analysis***. Reaction mixture was examined with an ACUITY UPLC H‐Class ultra‐performance liquid chromatography mass spectroscopy (UPLC–MS) system (Waters) equipped with a reverse‐phase columns column (Waters, ACQUITY UPLC BEH C18 1.7 um 2.1 mm×50 mm), an autosampler (Waters, SMFTN; 186015017), a pump (Waters, QSM; 186015018), a PDA detector (Waters, eλ Detector; 186015033), and an MS detector (Waters, QDa; 186006511), using 0.1% formate solution (solution A) and acetonitrile (solution B) as eluents. A/B=95/5 to 5/95 (0–3.5 min), 5/95 (3.5–4.0 min), 5/95 to 95/5 (4.0–4.1 min), 95/5 (4.1–5.0 min).

## Conflict of interest

The authors declare no conflict of interest.

1

## Supporting information

As a service to our authors and readers, this journal provides supporting information supplied by the authors. Such materials are peer reviewed and may be re‐organized for online delivery, but are not copy‐edited or typeset. Technical support issues arising from supporting information (other than missing files) should be addressed to the authors.

Supporting Information

## Data Availability

The data that support the findings of this study are available from the corresponding author upon reasonable request.
