# Supplementary material for: 9‐Cyano‐10‐telluriumpyronin Derivatives as Red‐light‐activatable Raman Probes
Source: Chem Asian J. 2022 Dec 13;18(2):e202201086. doi: 10.1002/asia.202201086 (PMC10107100; doi:10.1002/asia.202201086)
Supplement: Supplementary file 1 — Supporting Information [file ASIA-18-0-s001.pdf]

CHEMISTRY
AN ASIAN JOURNAL

Supporting Information

9-Cyano-10-telluriumpyronin Derivatives as Red-light-activatable Raman Probes

Minoru Kawatani, Spencer J. Spratt, Hiroyoshi Fujioka, Jingwen Shou, Yoshihiro Misawa, Ryosuke Kojima, Yasuteru Urano, Yasuyuki Ozeki, and Mako Kamiya*© 2022 The Authors. Chemistry - An Asian Journal published by Wiley-VCH GmbH. This is an open access article under the terms of the Creative Commons Attribution License, which permits use, distribution and reproduction in any medium, provided the original work is properly cited.

Safety statement.

No unexpected or unusual safety hazards were encountered.

Materials.

General chemicals were of the best grade available, supplied by Tokyo Chemical Industries, FUJIFILM Wako Pure Chemical or Aldrich Chemical Company, and were used without further purification.

Instruments.

Purification by column chromatography was performed on a YFLC-AI580 chromatograph (Yamazen). Preparative HPLC was performed on an HPLC system composed of reverse-phase columns of Inertsil ODS-3 10 mm × 250 mm or 20 mm × 250 mm (GL Sciences) with a pump (JASCO, PU-2087) and a detector (JASCO, MD-2015). Purification by gel permeation chromatography (GPC) was performed on a recycling preparative HPLC system (Japan Analytical Industry, LC-9210 NEXT) with a column (Japan Analytical Industry, JAIGEL 2HR column). ^{1}H NMR and ^{13}C NMR spectra were recorded on a Bruker AVANCEIII400 instrument (400 MHz for ^{1}H, 101 MHz for ^{13}C) with chemical shifts (δ) relative to residual solvent signals for ^{1}H and ^{13}C. High-resolution mass spectra were recorded on a Bruker micrOTOF II, using electrospray ionization (ESI). LC-MS analysis was performed on an ACQUITY UPLC system (Waters) composed of a BEH C18 1.7 μm 2.1 mm×50 mm column (Waters) with a QDa ESI-MS detector. Light irradiation was performed with 650 nm light from a xenon lamp (MAX303, Asahi Bunko, filtered through a 650/10 nm bandpass filter) All experiments were carried out at 298 K, unless otherwise specified.

Synthesis and characterization.

9CN-PY, 9CN-SiPY and 9CN-CPY were synthesized as previously described[1].

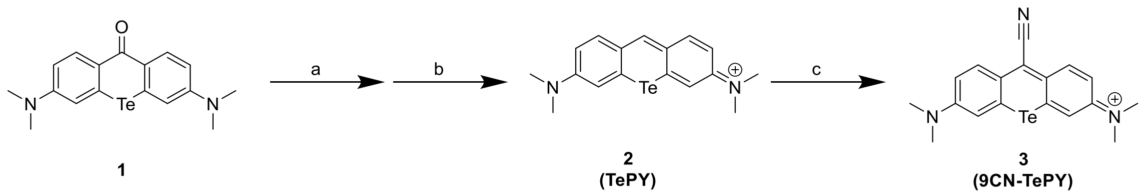

Scheme S1. Synthetic route to 9CN-TePY. Reagents and conditions: a) (i) lithium aluminum hydride ($LiAlH_4$), THF, 0 °C to 70 °C, (ii) HCl aq, 0 °C; b) *p*-chloranil, CH_2Cl_2, rt; c) (i) KCN, CH_3CN/water, rt, (ii) $FeCl_3$, HCl aq, rt.

Compound 2 (TePY).

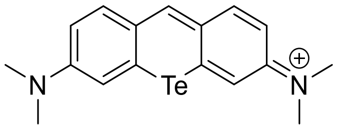

Compound **1** was synthesized according to the literature[2]. To a mixture of compound **1** (177.7 mg, 0.451 mmol) and THF (10 mL) was added $LiAlH_4$ (172.9 mg, 4.56 mmol, 10.1 eq). The resulting suspension was warmed to 70 °C and stirred for 18 hours. After cooling to room temperature, 2 mol/L HCl aq (6 mL) was added to quench the reaction, followed by addition of 10 mL water. The resulting mixture was extracted with CH_2Cl_2 (3 × 10 mL). The combined organic layer was washed with brine (30 mL), dried over anhydrous Na_2SO_4, and concentrated. The residue was subjected to silica gel flash chromatography (eluent: *n*-hexane/AcOEt = 91/9 to 70/30) to obtain a crude product (46.2 mg, 122 µmol). The crude product was dissolved in CH_2Cl_2 (2 mL). To this solution was added *p*-chloranil (30.3 mg, 123 µmol). The mixture was stirred for 35 min at room temperature, then sat. $NaHCO_3$ aq (10 mL) was added, followed by extraction with CH_2Cl_2 (5 × 20 mL). The combined organic layer was washed with brine (100 mL), dried over anhydrous Na_2SO_4, and concentrated. The residue was subjected to HPLC purification using eluent A (H_2O with 0.1% TFA) and eluent B (CH_3CN) (A/B = 90/10 to 0/100 for 40 min) to give the TFA salt of compound **2** as a purple solid (34.9 mg, 70.9 µmol, y. 16%). ^{1}H NMR (400 MHz, CD_3OD): δ=8.33 (s, 1H), 8.09 (d, J=9.4 Hz, 2H), 7.83 (d, J=2.5 Hz, 2H), 7.11 (dd, J=2.6 Hz, 9.3 Hz, 2H), 3.26 ppm (s, 12H); ^{13}C NMR (101 MHz, CD_3OD): δ=156.3, 154.5, 143.2, 139.8, 122.9, 116.7, 116.1, 40.7 ppm; HRMS (ESI) *m/z* calcd for $C_{17}H_{19}N_2Te^+$: 381.0606 $[M]^+$; found: 381.0604.

Compound 3 (9CN-TePY).

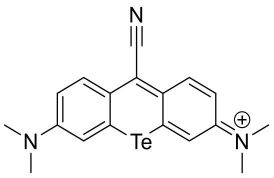

To a solution of compound **2** (42.3 mg, 86.0 µmol) in 2 mL CH_3CN and 0.2 mL water was added KCN (56.1 mg, 0.861 mmol, 10.0 eq), and the mixture was stirred for 15 min at room temperature. $FeCl_3 \cdot 6H_2O$ (115.5 mg, 0.427 mmol, 5.0 eq) solution in 0.8 mL 1 N HCl aq. Was added and stirring was continued for 90 min at room temperature. The reaction was quenched with sat. $NaHCO_3$ aq., and the suspension was filtered to obtain a crude product. This was purified by HPLC using eluent A (H_2O with 0.1% TFA) and eluent B (CH_3CN) (A/B = 90/10 to 0/100 for 40 min) to give the TFA salt of **9CN-TePY** as a purple solid (17.9 mg, 0.0346 mmol, y. 40%). ^{1}H NMR (400 MHz, CD_3CN): δ=8.49 (d, J=9.7 Hz, 2H), 7.75 (d, J=2.6 Hz, 2H), 7.18 (dd, J=2.5 Hz, 9.7 Hz, 2H), 3.24 (s, 12H)

ppm; ^{13}C NMR (101 MHz, [D$_6$]DMSO): δ=152.0, 137.7, 137.5, 128.1, 120.4, 117.7, 117.0, 116.8, 40.5 ppm; HRMS (ESI) *m/z* calcd for $C_{18}H_{18}N_3Te^+$: 406.0558 [*M*]$^+$; found: 406.0568.

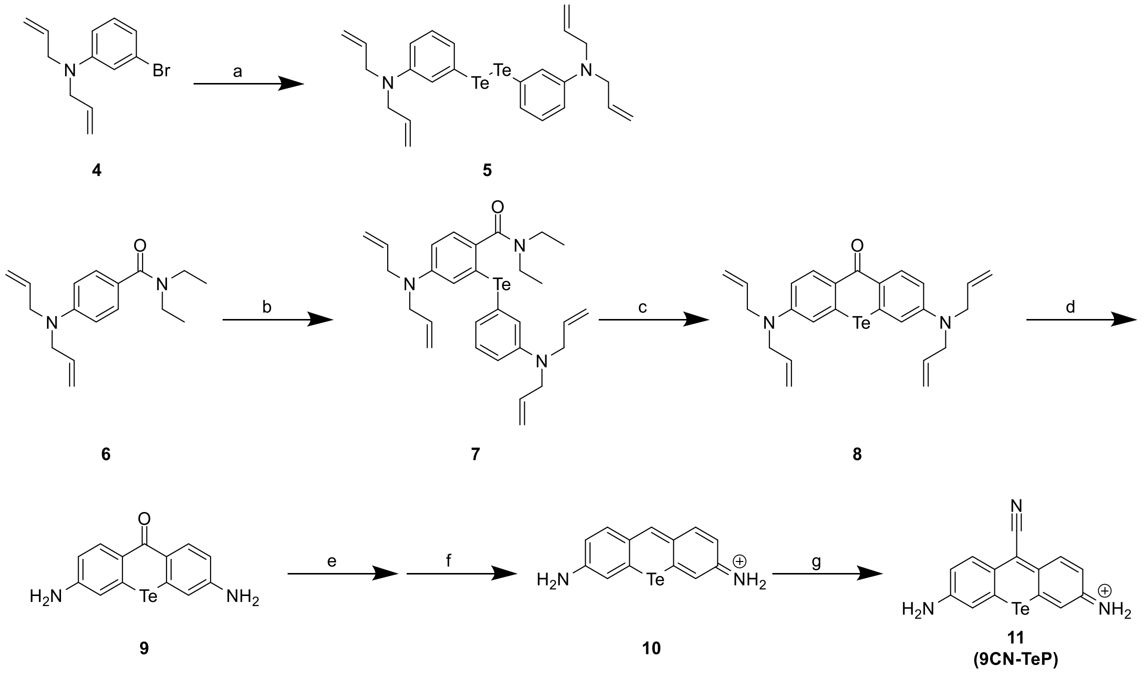

Scheme S2. Synthetic route to 9CN-TeP. Reagents and conditions: a) (i) Mg, THF, 0 °C to 45 °C, (ii) Te, 0 °C to rt, (iii) HCl aq, 0° C to rt; b) (i) *s*-BuLi, THF, -78 °C, (ii) **5**, THF, -78 °C to rt; c) (i) $POCl_3$, Et_3N, CH_3CN, 80 °C, (ii) NaOH aq, rt; d) 1,3-dimethylbarbituric acid, $Pd(PPh_3)_4$, CH_2Cl_2, 35 °C; e) (i) lithium aluminum hydride, THF, 65 °C, (ii) NaOH aq, rt; f) *p*-chloranil, CH_2Cl_2, rt; f) (g) KCN, CH_3CN/water, rt, (ii) $FeCl_3$, HCl aq, rt.

Compound 5.

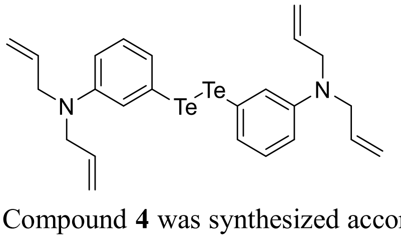

Compound **4** was synthesized according to the literature[3]. A solution of compound **4** (1712 mg, 6.8 mmol) in THF (5.5 mL) was added dropwise to ground Mg turnings (198 mg, 8.15 mmol, 1.2 eq). The mixture was stirred for 2 hours in room temperature, then heated at 45 °C for 1.5 hours under an Ar atmosphere. To the reaction mixture was added elemental Te (1.04 g, 8.15 mmol, 1.2 eq) at 0 °C, and stirring was continued at room temperature for 14 h under an Ar atmosphere. Then 1 M HCl aq (5 mL) and water (50 mL) were added and the mixture was stirred for 2.5 hours under air, and poured into 100 mL Et_2O. This suspension was filtered through a pad of Celite, and the organic layer of the filtrate was recovered. The aqueous layer was further extracted twice with 30 mL Et_2O. The combined extracts

were washed with 30 mL brine, dried over anhydrous Na_2SO_4, filtered and concentrated. The crude product was subjected to silica gel flash chromatography (eluent: *n*-hexane/AcOEt = 100/0 to 82/18), and to recycling preparative GPC to yield compound **5** as a yellow solid containing some impurity (777.1 mg, y. 19%, including the impurity). The purity was about 67% based on ^{1}H NMR analysis. The product was used for the next reaction without further purification.

Compound 7.

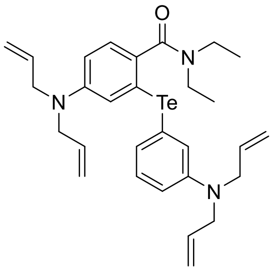

Compound **6** was synthesized according to the literature. Compound **6** (308.7 mg, 1.13 mmol) and *N,N,N',N'*-tetramethylethylenediamine (186 µL, 1.25 mmol, 1.1 eq) were dissolved in THF (15 mL) in a flask. The resulting solution was cooled to -78 °C and *sec*-butyllithium in THF (0.96 mL of a 1.3 M solution in cyclohexane-hexane solution, 1.25 mmol, 1.1 eq) was slowly added over 2 min under an Ar atmosphere. The mixture was stirred for 15 min at -78 °C and compound **5** (777 mg, 1.30 mmol, 1.1 eq) in THF (10 mL) was slowly added over 10 min to keep the temperature at -78 °C. The resulting mixture was warmed slowly from -78 °C to room temperature with stirring overnight. The reaction was then quenched with sat. NH_4Cl aq. (20 mL) and the whole was extracted with CH_2Cl_2 (1 × 40 mL and 2 × 30 mL). The organic extracts were combined, washed with 30 mL brine, dried over anhydrous Na_2SO_4, and concentrated. The crude product was subjected to silica gel flash chromatography (eluent: *n*-hexane/AcOEt = 100/0 to 50/50) to obtain compound **7** as a light-yellow oil (224.0 mg, 0.392 mmol, y. 35%). ^{1}H NMR (400 MHz, $CDCl_3$): δ=7.29–7.20 (m, 2H), 7.11–7.05 (m, 2H), 6.67 (dd, J = 2.4 Hz, 8.3 Hz, 1H), 6.58 (d, J = 2.5 Hz, 1H), 6.40 (dd, J = 2.5 Hz, 8.6 Hz, 1H), 5.87–5.77 (m, 2H), 5.66–5.64 (m, 2H), 5.17–5.12 (m, 4H), 5.02 (dd, J = 1.2, 10.3 Hz, 2H), 4.95 (dd, J = 1.32, 17.1 Hz, 2H), 3.89 (d, J = 4.8 Hz, 4H), 3.69 (d, J = 4.7 Hz, 4H), 3.47 (q, J = 7.1 Hz, 4H), 1.23 (t, J = 7.1 Hz, 6H) ppm; ^{13}C NMR (101 MHz, $CDCl_3$): δ=172.2, 149.3, 149.3, 133.7, 133.0, 129.9, 129.0, 127.8, 125.8, 124.9, 122.8, 118.3, 117.6, 116.2, 116.1, 112.4, 108.9, 52.6, 52.5, 41.9, 13.8 ppm; HRMS (ESI) *m/z* calcd for $C_{29}H_{38}N_3OTe^+$: 574.2074 [M+H]$^+$; found: 574.2092.

Compound 8.

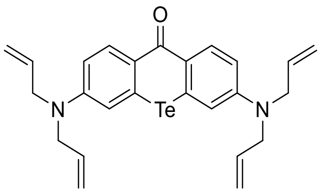

Compound **7** (85.9 mg, 0.172 mmol), and triethylamine (537 µL, 3.85 mmol, 10 eq) were dissolved in 10 mL CH_3CN. To the resulting solution was added dropwise $POCl_3$ (360 µL, 3.85 mmol, 10 eq) at 0 °C. The reaction solution was stirred for 1.5 hours at 80 °C and cooled to 0 °C again, followed by addition of 2 M NaOH aq (20 mL). The mixture was stirred for 1.5 hours at 0 °C and then extracted with CH_2Cl_2 (4 × 50 mL). The organic extracts were combined, washed with 10 mL brine, dried over anhydrous Na_2SO_4, and concentrated. The crude product was purified by silica gel flash chromatography (eluent: *n*-hexane/AcOEt = 82/18 to 61/39) to obtain compound **8** as a light-yellow solid (173.0 mg, 0.347 mmol, y. 90%). ^1H NMR (400 MHz, $CDCl_3$): δ=8.50 (d, J=9.2 Hz, 2H), 6.76 (d, J=2.1 Hz, 2H), 6.70 (dd, J=2.1 Hz, 9.2 Hz, 2H), 5.86–5.78 (m, 4H), 5.20–5.13 (m, 8H), 3.96–3.95 (m, 8H) ppm; ^{13}C NMR (101 MHz, $CDCl_3$): δ=183.4, 15.03, 133.9, 132.5, 123.7, 121.5, 116.6, 114.0, 112.3, 52.4 ppm; HRMS (ESI) *m/z* calcd for $C_{25}H_{27}N_2OTe^+$: 501.1182 $[M+H]^+$; found: 501.1180.

Compound 9.

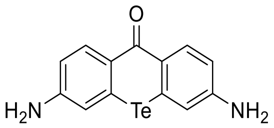

Compound **8** (85.9 mg, 0.172 mmol), tetrakis(triphenylphosphine)palladium (29.9 mg, 25.9 µmol, 0.15 eq) and 1,3-dimethylbarbituric acid (405 mg, 2.59 mmol, 15 eq) were dissolved in 10 mL THF. The resulting solution was warmed to 55 °C and stirred for 18.5 hours., then sat. $NaHCO_3$ aq (20 mL) was added and the mixture was filtered. The solid was extracted with methanol to recover the starting material, while the filtrate was extracted with CH_2Cl_2 (2 × 30 mL). The combined organic solution was washed with brine (10 mL), dried over anhydrous Na_2SO_4 and concentrated. The residue was purified by silica gel flash chromatography (eluent: *n*-hexane/AcOEt = 43/57 to 22/78) to obtain a crude product containing compound **9** as a light-red solid (52.0 mg, 154 µmol). HRMS (ESI) *m/z* calcd for $C_{13}H_{11}N_2OTe^+$: 340.9927 $[M+H]^+$; found: 340.9929. The crude product was directly used for the next reaction.

Compound 10.

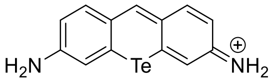

Crude **9** (52.0 mg, 154 μmol) was dissolved in 5 mL THF. To this solution was added $LiAlH_4$ (40.9 mg, 1.07 mmol, 7.0 eq) at 0 °C. The resulting suspension was warmed to 60 °C and stirred for 10 min. After cooling to 0 °C, 1 mol/L NaOH aq (1 mL) was added to quench the reaction, followed by addition of 20 mL water and 20 mL AcOEt. The reaction mixture was filtered through a Celite pad, and the organic layer was separated. The aqueous solution was further extracted with AcOEt (2 × 30 mL). The combined organic layer was washed with brine (10 mL), dried over anhydrous Na_2SO_4, and concentrated. To a solution of the crude product in CH_2Cl_2 (5 mL) was added *p*-chloranil (45.4 mg, 0.185 mmol, 1.2 eq). The reaction solution was stirred for 40 min at room temperature, washed with MeOH and concentrated. The residue was purified by HPLC using eluent A (H_2O with 0.1% TFA) and eluent B (CH_3CN) (A/B = 90/10 to 0/100 for 40 min) to give the TFA salt of compound **10** as a red solid (17.4 mg, 39.9 μmol, y. 23% from compound **8**). ^{1}H NMR (400 MHz, CD_3OD): δ=8.33 (s, 1H), 8.07 (d, J=9.0 Hz, 2H), 7.55 (d, J=2.2 Hz, 2H) 6.93 (dd, J=2.2 Hz, 9.0 Hz, 2H) ppm; ^{13}C NMR (101 MHz, CD_3OD): δ=157.1, 157.0, 144.6, 140.0, 122.9, 118.5, 118.2 ppm; HRMS (ESI) *m/z* calcd for $C_{13}H_{11}N_2Te^+$: 324.9979 [*M*]$^+$; found: 324.9971.

Compound 11 (9CN-TeP).

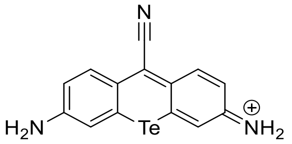

To a solution of compound **10** (17.4 mg, 39.9 μmol) in 7 mL CH_3CN were added 1 mol/L KCN aq (540 μL, 540 μmol, 10.0 eq), 1 mL water and 0.2 mL DMF. The mixture was stirred for 1.5 hours at room temperature, followed by 45 min at 40°C, and then 1 mol/L $FeCl_3$ in 1 mol/L HCl aq (27 μL, 27 μmol, 0.5 eq) was added. Stirring was continued for 2 min at room temperature, then the mixture was poured into water containing 1% acetonitrile and 0.1% TFA to purify by HPLC using eluent A (H_2O with 0.1% TFA) and eluent B (CH_3CN) (A/B = 90/10 to 0/100 for 40 min) to give the TFA salt of **9CN-TeP** as a purple solid (9.0 mg, 11.4 μmol, y. 49%). ^{1}H NMR (400 MHz, CD_3OD): δ=8.41 (d, J=9.4 Hz, 2H), 7.53 (d, J=2.0 Hz, 2H), 6.93 (dd, J=2.1 Hz, 9.4 Hz, 2H) ppm; ^{13}C NMR (101 MHz, CD_3OD): δ=156.4, 140.8, 140.0, 131.8, 122.1, 120.8, 119.0, 117.5 ppm; HRMS (ESI) *m/z* calcd for $C_{14}H_{10}N_3Te^+$: 349.9932 [*M*]$^+$; found: 349.9928.

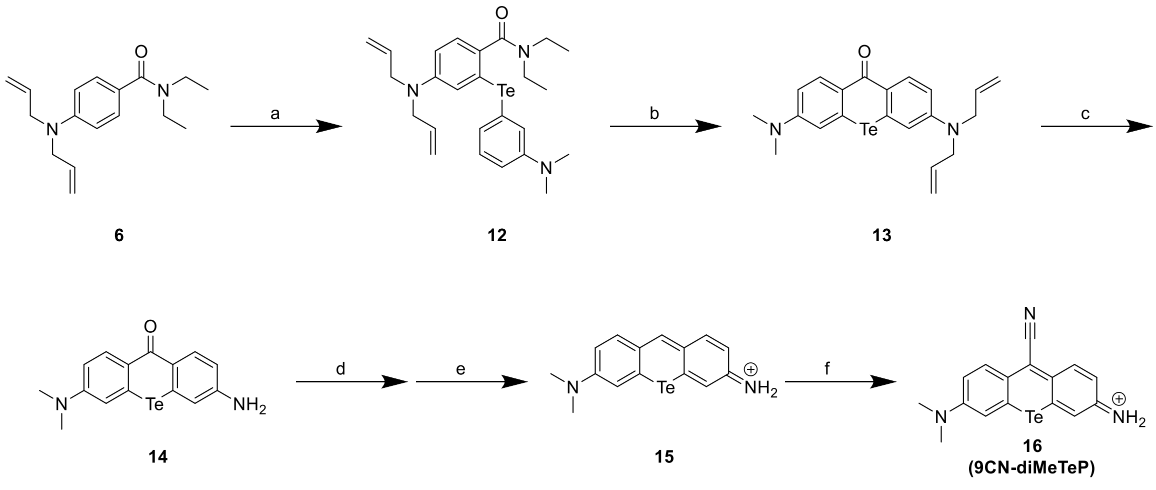

Scheme S3. Synthetic route to 9CN-diMeTeP. Reagents and conditions: a) (i) *s*-BuLi, THF, -78 °C, (ii) Di-3-*N,N*-dimethylaminophenyl ditelluride, THF, -78 °C to rt; b) (i) $POCl_3$, Et_3N, CH_3CN, 80 °C, (ii) NaOH aq, rt; c) 1,3-dimethylbarbituric acid, $Pd(PPh_3)_4$, CH_2Cl_2, 35 °C; d) (i) lithium aluminum hydride, THF, 65 °C, (ii) NaOH aq, rt; e) *p*-chloranil, CH_2Cl_2, rt; f) (i) KCN, CH_3CN/water, rt, (ii) $FeCl_3$, HCl aq, rt.

Compound 12.

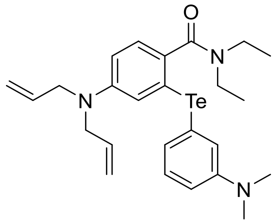

Di-3-*N,N*-dimethylaminophenyl ditelluride was synthesized according to the literature[4]. Compound **6** (2.114 g, 7.76 mmol) was dissolved in THF (15 mL) under an Ar atmosphere. The resulting solution was cooled to -78 °C and *sec*-butyllithium in THF (6.5 mL of a 1.3 M solution in cyclohexane-hexane solution, 8.40 mmol, 1.1 eq) was slowly added over 15 min. The mixture was stirred for 35 min at -78 °C and di-3-*N,N*-dimethylaminophenyl ditelluride (3.8533 g, 7.78 mmol, 1.1 eq) in THF (15 mL) was slowly added over 10 min to keep the temperature at -78 °C. The resulting mixture was warmed slowly from -78 °C to room temperature with stirring over 3 hours. The reaction was then quenched with 0.72 M NaH_2PO_4 aq (30 mL) and the mixture was extracted with AcOEt (3 × 20 mL). The organic extracts were combined, washed with 50 mL brine, dried over anhydrous Na_2SO_4, and concentrated. The crude product was subjected to silica gel flash chromatography (eluent: *n*-hexane/AcOEt = 96/4 to 20/80) to obtain compound **12** as a light-yellow oil (762.3 mg, 1.47 mmol, y. 19%). ^{1}H NMR (400 MHz, $CDCl_3$): δ=7.32–7.31 (m, 1H), 7.27–7.25 (m, 1H), 7.14–7.10 (m, 2H), 6.72 (ddd, *J*=0.75, 2.7, 8.4 Hz, 1H), 6.58 (d, *J*=2.6 Hz, 1H), 6.39 (dd, *J*=2.6, 8.6 Hz, 1H), 5.65–5.56 (m, 2H), 5.02–4.92 (m,

4H), 3.69–3.68 (m, 4H), 3.47 (q, *J*=7.1 Hz, 4H), 1.23 (t, *J*=7.1 Hz, 6H) ppm; ^{13}C NMR (101 MHz, $CDCl_3$): δ=172.3, 151.3, 149.4, 133.2, 129.9, 129.5, 128.0, 125.8, 125.5, 123.0, 118.2, 117.7, 116.2, 112.8, 108.9, 52.7, 42.0, 40.7, 13.9 ppm; HRMS (ESI) *m/z* calcd for $C_{25}H_{34}N_3OTe^+$: 522.1760 [*M*+H]$^+$; found, 522.1758.

Compound 13.

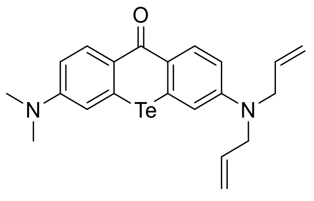

Compound **12** (746.9 mg, 1.44 mmol) was dissolved in 15 mL CH_3CN. To the resulting solution were added $POCl_3$ (1.34 mL, 14.8 mmol, 10 eq) and Et_3N (2.01 mL, 14.5 mmol, 10 eq). The reaction solution was stirred for 1 hour at 80 °C and cooled to room temperature, followed by dropwise addition of 2 M NaOH aq (24 mL). The mixture was stirred for 20 min. The precipitate was collected by filtration to obtain compound **13** as a yellow solid (535.3 mg, 1.20 mmol, y. 83%).^{1}H NMR (400 MHz, $CDCl_3$): δ=8.55-8.51 (m, 2H), 6.78-6.77 (m, 2H), 6.74–6.71 (m, 2H), 5.90–5.81 (m, 2H), 5.22–5.15 (m, 4H), 3.99–3.98 (m, 4H) 3.06 (s, 6H) ppm; ^{13}C NMR (101 MHz, $CDCl_3$): δ=183.7, 151.6, 150.4, 134.1, 134.0, 132.6, 123.9, 123.5, 121.7, 121.6, 116.8, 114.2, 114.0, 112.4, 112.1, 52.5, 40.1 ppm; HRMS (ESI) *m/z* calcd for $C_{21}H_{23}N_2OTe^+$: 449.0868 [*M*+H]$^+$; found, 449.0850.

Compound 14.

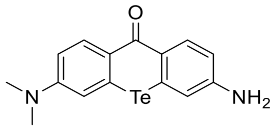

Compound **13** (250.7 mg 0.562 mmol), tetrakis(triphenylphosphine)palladium (66.0 mg, 57.1 µmol, 0.10 eq) and 1,3-dimethylbarbituric acid (1.0688 g, 6.85 mmol, 12.2 eq) were dissolved in 10 mL CH_2Cl_2. The resulting solution was warmed to 35 °C and stirred for 18 hours. The reaction mixture was filtered and the insoluble material was dried *in vacuo* to obtain compound **14** as a yellow solid (157.0 mg, 42.9 µmol, y. 76%). ^{1}H NMR (400 MHz, [D$_6$]DMSO): δ=8.29 (d, *J*=9.2 Hz, 1H), 8.20, (d, *J*=8.8 Hz, 1H), 7.13 (d, *J*=2.6 Hz, 1H), 6.88 (d, *J*=2.3 Hz, 1H), 6.77 (dd, *J*=2.7, 9.2 Hz, 1H), 6.58 (dd, *J*=2.3, 8.8 Hz, 1H), 6.03 (s, 2H), 3.01 (s, 6H) ppm; ^{13}C NMR (101 MHz, [D$_6$]DMSO): δ=181.9, 151.5, 151.2, 133.3, 132.8, 122.1, 122.1, 122.0, 121.7, 115.4, 114.1, 113.8, 111.6, 40.2 ppm; HRMS (ESI) *m/z* calcd for $C_{15}H_{15}N_2OTe^+$: 369.0242 [*M*+H]$^+$; found: 369.0235.

Compound 15.

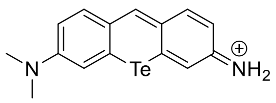

To a mixture of compound **14** (147.8 mg, 0.404 mmol) and THF (10 mL) was added $LiAlH_4$ (107.1 mg, 2.82 mmol, 7.0 eq). The resulting suspension was warmed to 65 °C and stirred for 1 hour. After cooling to room temperature, 2 mol/L NaOH aq (1 mL) was added slowly to quench the reaction, followed by addition of 9 mL water and 8 mL AcOEt, and the organic layer was recovered. The aqueous solution was further extracted with AcOEt (2 × 8 mL). The combined organic layer was washed with brine (10 mL), dried over anhydrous Na_2SO_4, and concentrated to obtain a crude product. To a solution of the crude product (142.2 mg, 0.404 mmol) in CH_2Cl_2 (8 mL) was added *p*-chloranil (112.5 mg, 0.459 mmol, 1.1 eq),. The mixture was stirred for 60 min at room temperature, and then sat. $NaHCO_3$ aq (10 mL) was added. The resulting mixture was extracted with CH_2Cl_2/MeOH (9:1) (3 × 10 mL). The organic extracts were combined, washed with 20 mL brine, dried over anhydrous Na_2SO_4, and concentrated. The residue was purified by HPLC using eluent A (H_2O with 0.1% TFA) and eluent B (CH_3CN) (A/B = 90/10 to 0/100 for 40 min) to give the TFA salt of **15** as a red solid (145.0 mg, 0.312 mmol, y. 77%).^{1}H NMR (400 MHz, CD_3OD): δ=8.34 (s, 1H), 8.11 (d, J=9.4 Hz, 1H), 8.06 (d, J=9.0 Hz, 1H), 7.84 (d, J=2.5 Hz, 1H), 7.54 (d, J=2.2 Hz, 1H), 7.13 (dd, J=2.6, 9.3 Hz, 1H), 6.91 (dd, J=2.2, 8.9 Hz, 1H), 3.27 (s, 6H) ppm; ^{13}C NMR (101 MHz, $CDCl_3$): δ=154.3, 151.2, 139.2, 138.8, 137.9, 137.4, 128.4, 120.4, 120.1, 119.8, 118.0, 117.5, 116.8, 116.7, 40.5; HRMS (ESI) *m/z* calcd for $C_{15}H_{15}N_2Te^+$: 353.0293 $[M]^+$; found: 353.0290.

Compound 16 (9CN-diMeTeP).

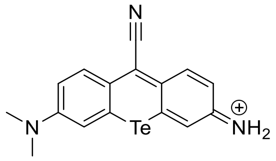

To a solution of compound **15** (72.6 mg, 156 μmol) in 2 mL CH_3CN and 0.2 mL H_2O was added KCN (107.5 mg, 1.65 mmol, 10.5 eq). The resulting mixture was stirred for 2 hours at room temperature, followed by addition of $FeCl_3$ in 1 mol/L HCl aq (219.4 mg, 1.5 mL, 812 μmol, 5.2 eq). Stirring was continued for 50 min at room temperature, then the reaction was quenched by addition of 20 mL sat. $NaHCO_3$ aq. The insoluble material was collected by filtration to obtain a crude product, which was purified by HPLC using eluent A (H_2O with 0.1% TFA) and eluent B (CH_3CN) (A/B = 90/10 to 0/100 for 40 min) to give the TFA salt of **9CN-diMeTeP** as a purple solid (19.2 mg, 39.3 μmol, y. 25%).^{1}H NMR (400 MHz, CD_3OD): δ=8.61 (d, J=9.8 Hz, 1H), 8.56 (d, J=9.4 Hz, 1H), 8.02 (d, J=2.7 Hz, 1H), 7.67 (d, J=2.3 Hz, 1H), 7.33 (dd, J=2.7, 9.7 Hz, 1H), 7.06 (dd, J=2.3, 9.4 Hz, 1H), 3.32 (s, 6H) ppm;

HRMS (ESI) *m/z* calcd for $C_{16}H_{14}N_3Te^+$: 378.0245 [*M*]$^+$; found: 378.0235.

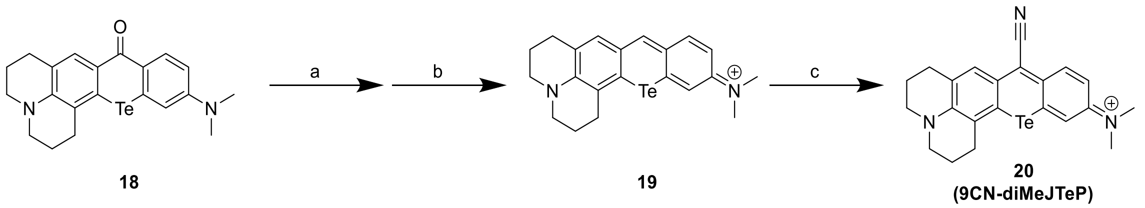

Scheme S4. Synthetic route to 9CN-diMeJTeP. Reagents and conditions: a) (i) lithium aluminum hydride, THF, 65 °C, (ii) NaOH aq, rt; b) *p*-chloranil, CH_2Cl_2, rt; c) (i) KCN, CH_3CN/water, rt, (ii) $FeCl_3$, HCl aq, rt.

Compound 19.

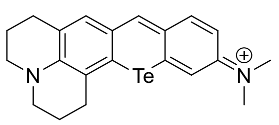

Compound **18** was synthesized according to the literature[5]. Compound **18** (30.9 mg, 69 µmol) was dissolved in 5 mL THF and to the solution was added $LiAlH_4$ (18.4 mg, 485 µmol, 7.0 eq) on ice. The resulting suspension was warmed to 60 °C, stirred for 30 min under an Ar atmosphere, and then cooled on ice. To the mixture was slowly added 1 mol/L NaOH aq (1 mL) to quench the reaction, followed by addition of 20 mL water and 20 mL AcOEt. The whole was filtered through a Celite pad, and the organic layer was recovered from the filtrate. The aqueous solution was further extracted with AcOEt (1 × 50 mL). The combined organic layer was washed with brine (10 mL), dried over anhydrous Na_2SO_4, and concentrated to obtain a crude product. To a solution of the crude product in CH_2Cl_2 (5 mL) was added *p*-chloranil (13.6 mg, 0.055 mmol, 0.8 eq). The reaction mixture was stirred for 20 min at room temperature and then directly subjected to silica gel flash chromatography (eluent: CH_2Cl_2/MeOH = 100/0 to 80/20) to obtain compound **19** as a red solid (16.3 mg, 37.8 µmol, y. 55%). ^{1}H NMR (400 MHz, CD_3OD): δ=8.07 (s, 1H), 7.99 (d, *J*=9.2 Hz, 1H), 7.73 (d, *J*=2.2 Hz, 1H), 7.69 (s, 1H), 7.04 (dd, *J*=2.3, 9.2 Hz, 1H), 3.53–3.49 (m, 4H), 3.20 (s, 6H), 2.88–2.85 (m, 2H), 2.57-2.54 (m, 2H), 2.16–2.10 (m, 2H), 2.03–1.97 (m, 2H) ppm; HRMS (ESI) *m/z* calcd for $C_{16}H_{14}N_3Te^+$: 433.0919 [M]$^+$; found: 433.0925.

Compound 20 (9CN-diMeJTeP).

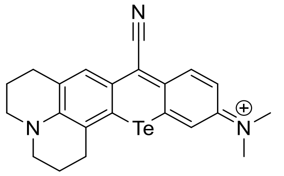

To a solution of compound **19** (16.3 mg, 37.8 μmol) in 5 mL CH_3CN were added KCN (107.5 mg, 1.65 mmol, 4.5 eq) and 2 mL water. The resulting mixture was stirred for 25 min at room temperature, followed by addition of $FeCl_3$ in 1 mol/L HCl aq (18.9 μL, 18.9 μmol, 0.5 eq), and stirring was continued for 10 min at room temperature. The reaction mixture was directly purified by HPLC using eluent A (H_2O with 0.1% TFA) and eluent B (CH_3CN) (A/B = 90/10 to 0/100 for 40 min) to give the TFA salt of **9CN-diMeJTeP** as a red solid (8.1 mg, 14.2 μmol, y. 38%). ^{1}H NMR (400 MHz, CD_3OD): δ=8.29 (d, J=9.6 Hz, 1H), 8.01 (s, 1H), 7.74 (d, J=2.5 Hz, 1H), 7.11 (dd, J=2.4, 9.6 Hz, 1H), 3.50 (br, 4H), 3.16 (s, 6H), 2.87 (dd, J=5.9, 5.9 Hz, 2H), 2.43 (dd, J=6.4, 6.4 Hz, 2H), 2.11–2.09 (m, 2H), 2.00–1.97 (m, 2H) ppm; ^{13}C NMR (101 MHz, CD_3OD): δ=153.3, 150.4, 138.2, 137.0, 136.6, 135.3, 129.9, 128.7, 124.7, 123.5, 120.7, 117.8, 117.5, 116.8, 53.1, 52.4, 40.8, 31.3, 28.4, 21.4, 21.2 ppm; HRMS (ESI) m/z calcd for $C_{22}H_{22}N_3Te^+$: 458.0872 $[M]^+$; found: 458.0856.

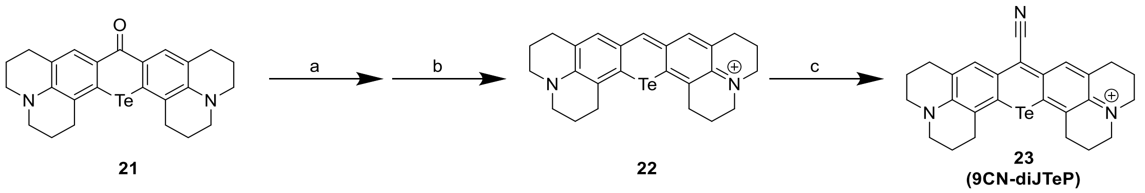

Scheme S5. Synthetic route to 9CN-diJTeP. Reagents and conditions: a) (i) lithium aluminum hydride, THF, 65 °C, (ii) NaOH aq, rt; b) *p*-chloranil, CH_2Cl_2, rt; c) (i) KCN, CH_3CN/water, rt, (ii) $FeCl_3$, HCl aq, rt.

Compound 22.

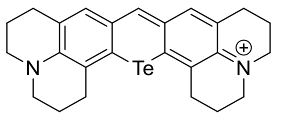

Compound **21** was synthesized according to the literature[6]. Compound **21** (20 mg, 40 μmol) was dissolved in 8 mL THF and to the solution was added $LiAlH_4$ (10.7 mg, 281 μmol, 7.0 eq) on ice. The resulting suspension was warmed to 60 °C and stirred for 30 min under an Ar atmosphere. After cooling on ice, 1 mol/L NaOH aq (0.1 mL) was added slowly to quench the reaction, followed by addition of 10 mL water and 30 mL AcOEt. The whole was filtered through a Celite pad, and the organic layer was recovered from the filtrate. The aqueous solution was further extracted with AcOEt (3 × 20 mL). The combined organic layer was washed with brine (20 mL), dried over anhydrous

Na_2SO_4, and concentrated to obtain a crude product. To a solution of the crude product in dichloromethane (1 mL) and MeOH (1 mL) was added *p*-chloranil (9.9 mg, 40 µmol, 1.0 eq). The reaction mixture was stirred for 5 min at room temperature and directly subjected to silica gel flash chromatography (eluent: dichloromethane/MeOH = 100/0 to 80/20) to obtain compound **22** as a blue solid (17.0 mg, 35.2 µmol, y. 88%). ^{1}H NMR (400 MHz, CD_3OD): δ=7.93 (s, 1H), 7.62 (s, 2H), 3.43 (dd, J=5.7, 5.7 Hz, 8H), 2.81 (dd, J=6.2, 6.2 Hz, 4H), 2.60 (dd, J=6.4, 6.4 Hz, 4H), 2.10–2.04 (m, 4H), 1.96–1.90 (m, 4H) ppm; ^{13}C NMR (101 MHz, CD_3OD): δ=154.0, 149.6, 139.8, 135.3, 126.0, 122.7, 122.3, 52.2, 51.6, 31.1, 28.3, 21.7, 21.6 ppm; HRMS (ESI) *m/z* calcd for $C_{25}H_{27}N_2Te^{+}$: 485.1223 $[M]^{+}$; found: 485.1233.

Compound 23 (9CN-diJTeP).

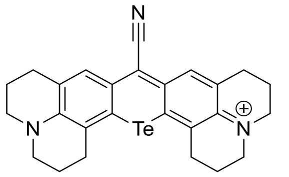

To a solution of compound **23** (7.5 mg, 16 µmol) in 5 mL CH_3CN and 1 mL water was added 1 mol/L KCN aq (109 µL, 109 µmol, 7 eq). The resulting mixture was stirred for 60 min at room temperature, followed by addition of $FeCl_3$ in 1 mol/L HCl aq (7.8 µL, 7.8 µmol, 0.5 eq) and 1 mol/L HCl aq, and stirring was continued for 5 min at room temperature. The reaction mixture was directly purified by HPLC using eluent A (H_2O with 0.1% TFA) and eluent B (CH_3CN) (A/B = 90/10 to 0/100 for 40 min) to give the TFA salt of **9CN-diJTeP** as a green solid (1.7 mg, 3.3 µmol, y. 22%). ^{1}H NMR (400 MHz, CD_3OD): δ=8.00 (s, 2H), 3.50-3.47 (m, 8H), 2.88 (dd, J=6.2, 6.2 Hz, 4H), 2.53 (dd, J=6.5, 6.5 Hz, 4H), 2.12–2.09 (m, 4H), 1.99–1.96 (m, 4H) ppm; ^{13}C NMR (101 MHz, CD_3OD): δ=149.4, 135.8, 134.0, 128.7, 128.0, 123.6, 121.7, 118.2, 52.7, 51.9, 31.4, 28.6, 21.5 ppm. HRMS (ESI) *m/z* calcd for $C_{26}H_{26}N_3Te^{+}$: 510.1185 $[M]^{+}$; found: 510.1191.

Supplementary Figures.

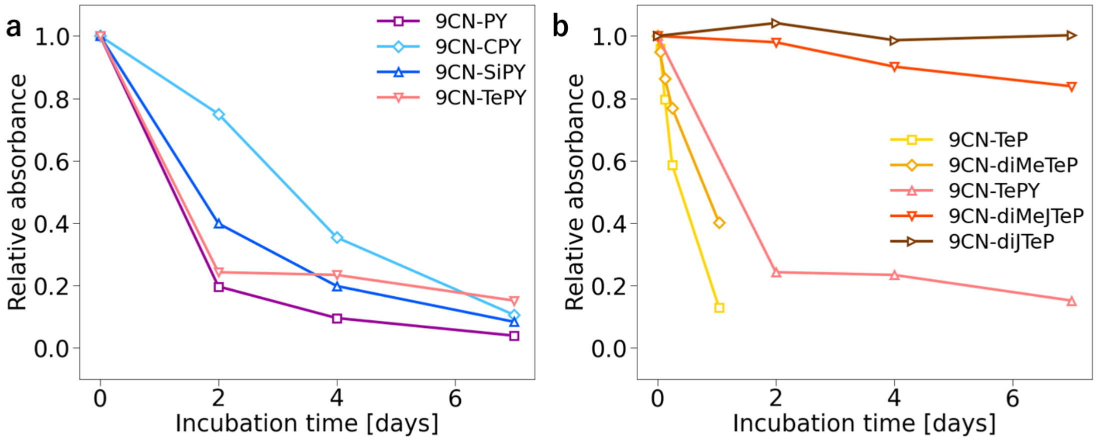

Figure S1. Stability of 9-cyanopyronin derivatives. (a) 9CN-PY derivatives and (b) 9CN-TeP derivatives are compared. Solvent: PBS (1% DMSO (9CN-PY, 9CN-CPY and 9CN-SiPY or 1% DMF (other dyes) was added as a cosolvent), dye concentration: 10 µM. Temperature: 37 °C. Samples were stored in the dark. For each time point, 200 µL of each sample was diluted to 2.5 mL PBS for measurement of the absorption spectra. Maximum absorption intensity was plotted over a day (9CN-TeP and 9CN-diMeTeP) or a week (other dyes).

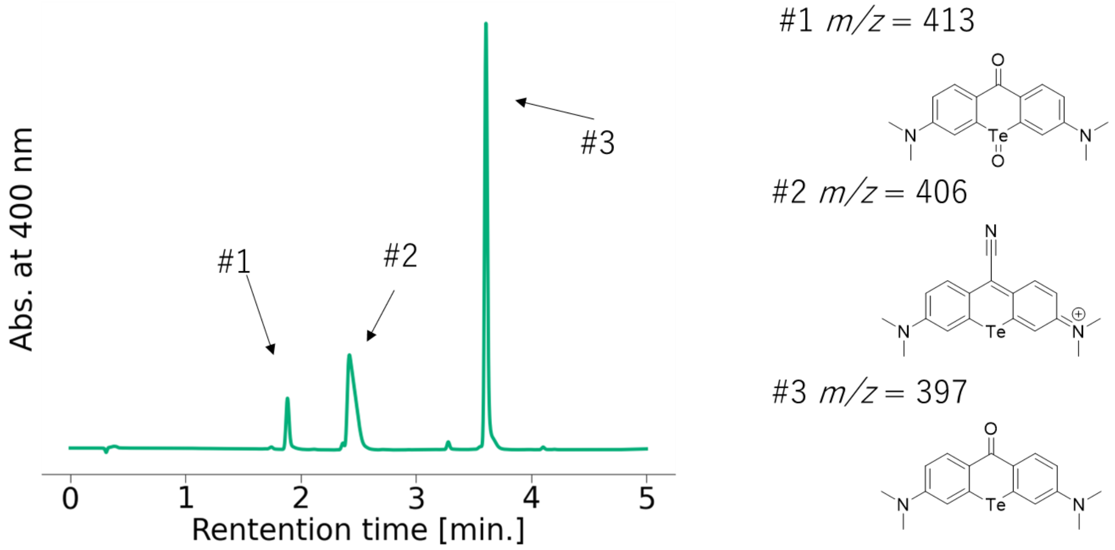

Figure S2. LC-MS analysis of the oxidation product of 9CN-TePY. Absorbance at 400 nm was monitored. Solvent: PBS, dye concentration: 100 µM, NaOCl concentration: 100 µM. 50% DMF was added as cosolvent.

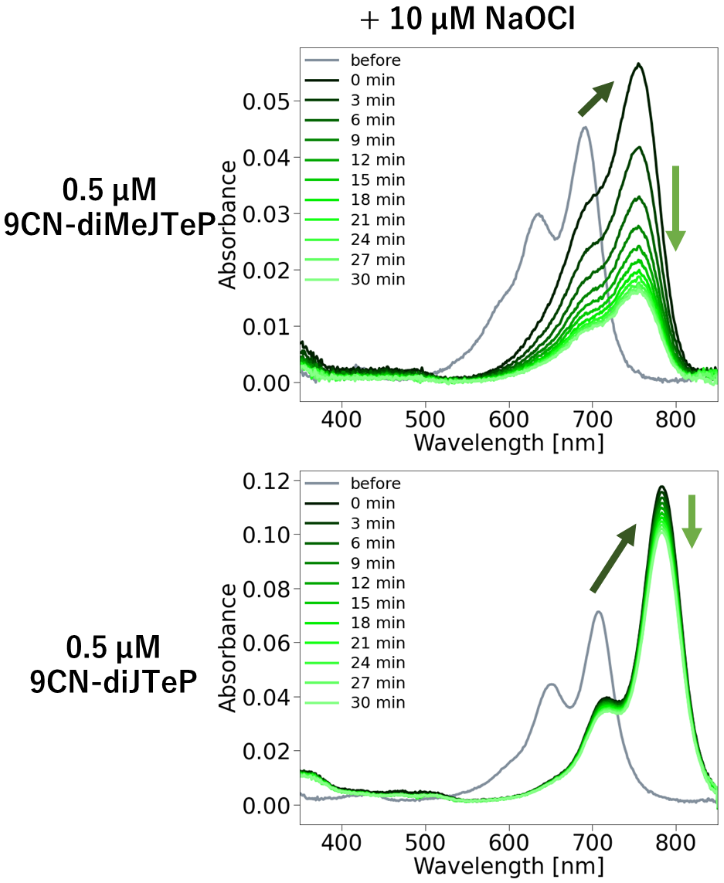

Figure S3. Change in absorption spectra of 9CN-diMeJTeP and 9CN-diJTeP after oxidation by NaOCl. Solvent: PBS, dye concentration: 0.5 μM (9CN-diMeJTeP and 9CN-diJTeP), NaOCl concentration: 10 μM. Absorption spectra were measured before oxidation (before) and at 3 min intervals after addition (0–30 min).

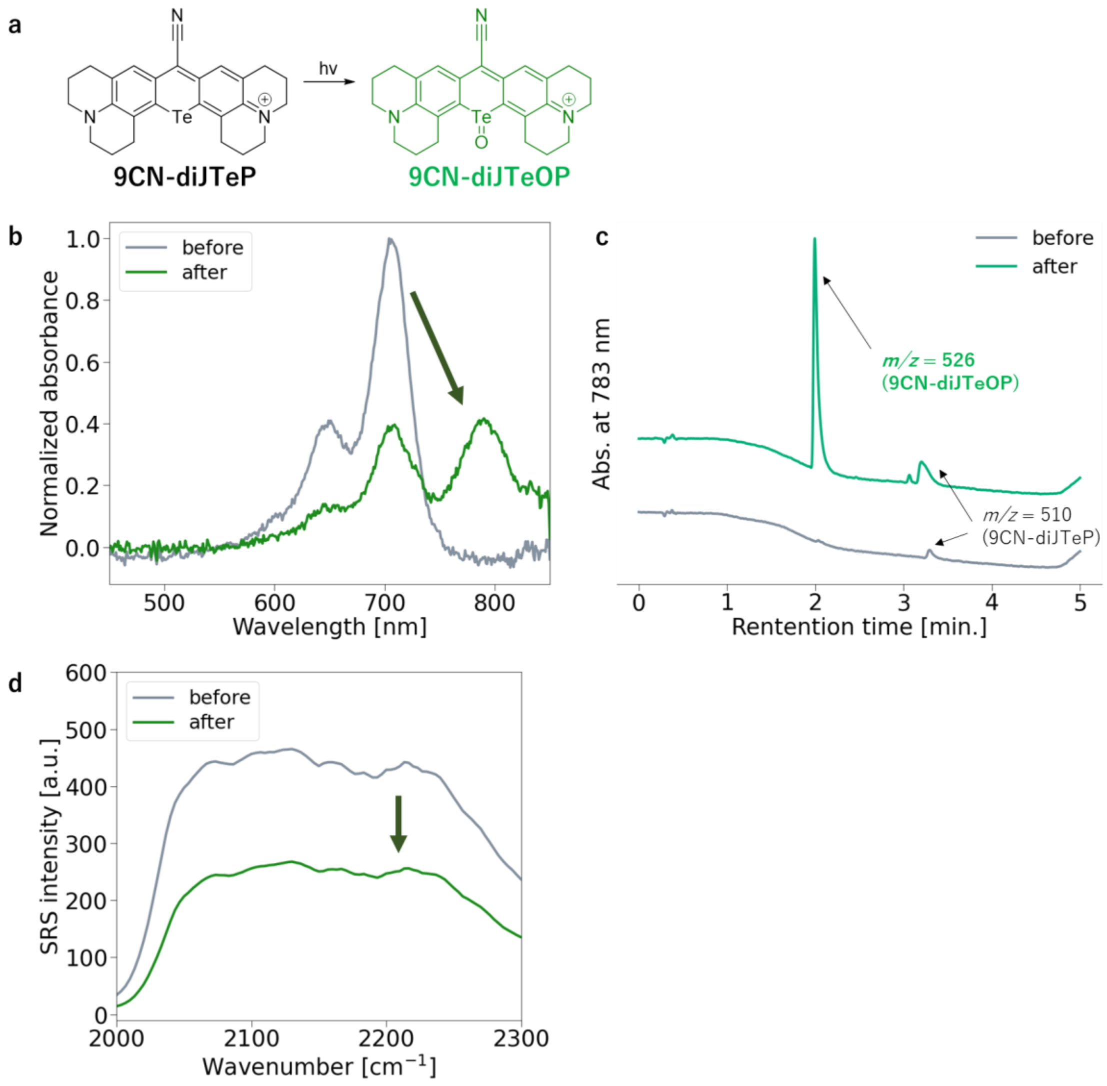

Figure S4. Photoreaction of 9CN-diJTeP. (a) Anticipated reaction scheme of photooxidation. (b) Change in absorption spectra upon photoirradiation. (c) LC-MS analysis of photoproduct. The absorbance at 783 nm was monitored. (d) Change in SRS spectra upon photoirradiation. Solvent: 20 mM sodium phosphate buffer at pH 2.0, concentration of dye for photoreaction: 100 µM, DMF: 10%. Absorption spectra were measured by diluting 10 µL reaction solution to 2.5 mL with 20 mM sodium phosphate buffer at pH 2.0 (final concentration: 0.4 µM). Photoreaction: 45 mW/cm^2, xenon light through a 650/10 nm bandpass filter.

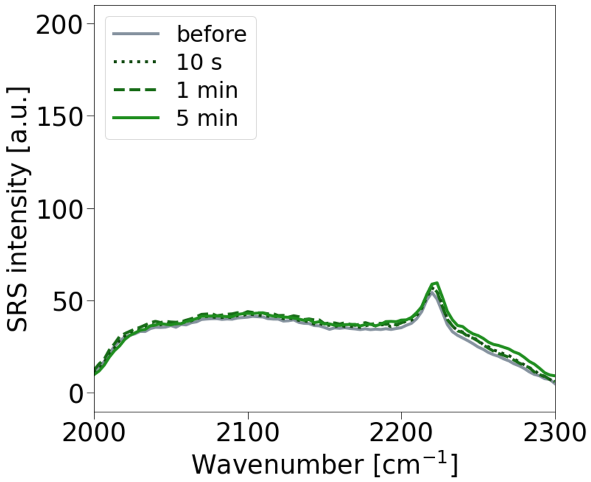

Figure S5. Change in SRS spectrum of 9CN-diMeJTeP during observation without 640 nm light irradiation. Solvent: 20 mM sodium phosphate buffer at pH 2.0, concentration of dye for photoreaction: 100 μM, DMF: 10%. Laser intensity: 20 mW. Note that since the scanning volume was very small compared with the total volume of the mounted solution, it is difficult to exclude the possibility that some molecules activated by the SRS laser might diffuse out of scanning laser spot and thus not be detected.

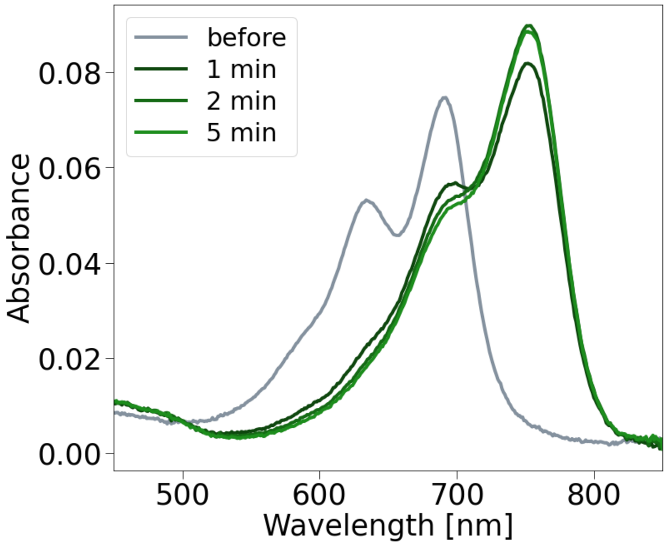

Figure S6. Stability of 9CN-diMeJTeP after photooxidation in neutral buffer. 1 μM 9CN-diMeJTeP solution in PBS was prepared in a transparent cuvette. After acquiring “before” spectra, the cuvette was irradiated for 1 minute with 650 nm light, followed by spectral observation (“1 min” data). The cuvette was similarly irradiated for another 1 minute before taking “2 min” data, and for a further 3 minutes before taking “5 min” data. Light source: 45 mW/cm^2, xenon light through a 650/10 nm bandpass filter.

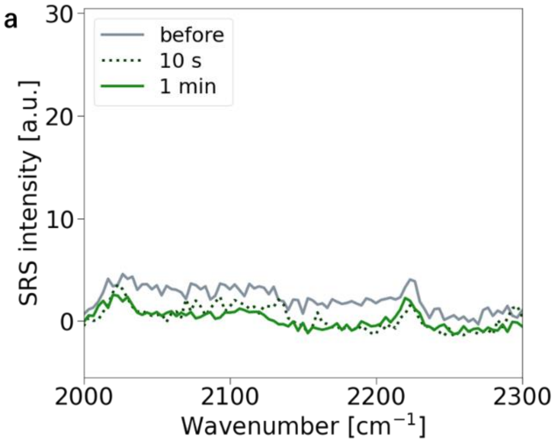

Figure S7. *In situ* photoactivation of 9CN-diMeJTeP in neutral buffer on an SRS microscope. Change in SRS spectra without (a) or with (b) 640 nm light irradiation. Solvent: PBS, concentration of dye: 10 μM, DMF: 10%. 640 nm light was continuously applied during observation. Laser intensity: 20 mW.

Supplementary references

[1] L. Wei, Z. Chen, L. Shi, R. Long, A. V. Anzalone, L. Zhang, F. Hu, R. Yuste, V. W. Cornish, W. Min, *Nature* **2017**, *544*, 465–470.

[2] B. Calitree, D. J. Donnelly, J. J. Holt, M. K. Gannon, C. L. Nygren, D. K. Sukumaran, J. Autschbach, M. R. Detty, *Organometallics* **2007**, *26*, 6248–6257.

[3] H. Fujioka, J. Shou, R. Kojima, Y. Urano, Y. Ozeki, M. Kamiya, *J. Am. Chem. Soc.* **2020**, *142*, 20701–20707.

[4] D. J. Del Valle, D. J. Donnelly, J. J. Holt, M. R. Detty, *Organometallics* **2005**, *24*, 3807–3810.

[5] J. J. Holt, B. D. Calitree, J. Vincek, M. K. Gannon, M. R. Detty, *J. Org. Chem.* **2007**, *72*, 2690–2693.

[6] M. W. Kryman, G. A. Schamerhorn, J. E. Hill, B. D. Calitree, K. S. Davies, M. K. Linder, T. Y. Ohulchanskyy, M. R. Detty, *Organometallics* **2014**, *33*, 2628–2640.
